# Dynamics of Neuropsychological Symptoms during the Training of Executive Functions in Neurological Patients

**DOI:** 10.3390/healthcare9121716

**Published:** 2021-12-13

**Authors:** Nikolai Shcherbakov, Nataliya Varako, Maria Kovyazina, Yulia Zueva, Maria Baulina, Anatoliy Skvortsov, Daria Chernikova

**Affiliations:** 1 Faculty of Psychology, Lomonosov Moscow State University, 119991 Moscow, Russia; nvarako@mail.ru (N.V.); kms130766@mail.ru (M.K.); psycho-sovet@mail.ru (M.B.); skwortsow@mail.ru (A.S.); lunadashache@gmail.com (D.C.); 2 Research Center of Neurology, 125367 Moscow, Russia; 3 Psychological Institute of the Russian Academy of Education, 125009 Moscow, Russia; 4 Moscow Center for Rehabilitation Treatment, 141407 Moscow, Russia; yzoueva@yandex.ru; 5 Faculty of Clinical Psychology, Moscow Institute of Psychoanalysis, 121170 Moscow, Russia

**Keywords:** executive disorder, frontal cortex, rehabilitation, group training, cognitive strategies

## Abstract

Executive function disorder rehabilitation in neurological patients is associated with many difficulties. We investigated the effectiveness of group training, proposed by B. Wilson et al., which has the model of frontal lobes functioning by D. T. Stuss as the theoretical background. The study participants were 16 patients with executive function disorder caused by TBI, strokes, and infections. The training was shortened from 9 weeks to 3 and adopted to the conditions of the rehabilitation centre where the study was held. The evaluation of training effectiveness was carried out by the methods of neuropsychological diagnostics proposed by A. R. Luria as well as standardized quantitative tests (CWIT test, Raven test, FAB) and questionnaires (EBIQ) aimed at assessing the state of executive functions and general well-being. In result positive trends, but not reaching the level of significance, were revealed in the performance of all evaluating methods, with the exception of “arithmetic problems” and “inhibitory control” as part of the FAB test. Statistically significant result was obtained concerning such tests as “counting”, “analysis of story pictures”, and index of total uncorrected errors in the CWIT test. Thus, the results of eventual assessment showed positive dynamic of executive functions state.

## 1. Introduction

Executive function disorder is one of the most prevalent consequences of brain damage. Executive function rehabilitation plays crucial role in patients’ quality of life, since this impairment affects functioning in everyday life, at work and communication with others [1]. In the methodology of psychology, this is reflected in the comparison of various paradigms of scientific knowledge [2]. Despite high level of interest in executive functions, there is a lack of research in this area: for example, there is a debate regarding which components of mental functions should be considered as “executive functions” [3,4].

One of the most well-developed executive functions theories is Stuss’s model which subdivides executive functions on four different domains that correspond to different anatomical regions of the frontal cortex. A briefly review of the functions of each of them described below [5].

*Energization* is related to the dorsomedial region of frontal cortex. In line with Stuss, impairment of this function is related to the processes of initiation and sustaining any response. Deficit of this function manifests itself in the lower speed of task progression, if the task requires speeded responses, as well as in the inability to focus attention and fatigue.

*Executive functions* are related to the dorsolateral prefrontal cortex and take part in two main regulatory functions: monitoring and task setting. Impairment of this domain manifests itself in difficulties of goal setting, programming, planning, and control on all levels of functioning, as well as in difficulties of choosing between alternatives [5].

*Behavioural/emotional self-regulation* is related to the ventromedial cortex (areas 32, 25, 24, 14, 13, 12, and 11). Impairments of this region cause behavioural difficulties, related to motivational, emotional, and social aspects. According to Stuss, standardized neuropsychological tests are not sensitive enough to detect these impairments. Behavioural difficulties manifest themselves in the situations which involve empathy, wilful deceit, and gambling. This domain is related to risk and rewards perception, as well as to expecting similar reactions from other people [5]. Thus, this function takes part in experiencing emotions adequate to the current situation, as well as demonstrating appropriate behavioural reactions.

*Metacognition/integration* is related to the frontal lobe (area 10, particularly in the right hemisphere). This function is integrative and coordinating. Processes of motivation, activation, emotional experience, regulatory functions necessary to solve complex and novel tasks: all interact in this function. Metacognitive functions contribute to our ability to be aware of our own point of view and point of view of other people, understand differences between them, being able to view the problem from different point of views and take situational factors into account [5].

Methods of executive function rehabilitation involve exercises to develop volitional attention, self-observation, skills of comparing and structuring information, planning, problem solving, and others and are well known in neuropsychological rehabilitation [6].

Application of cognitive strategies (deductive schemes of action, which involve several steps, adapted for regulatory problems in patient’s everyday life) can be a potential alternative to working on specific skills, associated with regulatory functions [7]. That is, these strategies use self-awareness and self-control as a main method to compensate executive functions impairment. This approach may allow patients to adapt to a broader spectrum of everyday life situations.

This approach to rehabilitation correlates with the principles proposed by G. P. Prigatano. Thus, Prigatano notes that the problem of violation of the patient’s self-awareness is often given insufficient attention during rehabilitation. Meanwhile, according to his opinion, neuropsychological rehabilitation should focus both on restoring higher mental functions and on ways of functioning in situations of interpersonal communication. Effective neuropsychological rehabilitation should help patients to see the peculiarities of their own behaviour and thus help them to realize the consequences of traumatic brain injury [8].

For example, Tornas and colleagues [9] described an approbation of Goal Management Training (GMT), based on the theory of executive functions [10]. Based on the above-described model of executive functions developed by Stuss [5], researchers emphasized the role of energization and executive functions in the rehabilitation. Researchers assumed that frontal lobes impairment causes patients to react on irrelevant signals in the outside world and inability to control these reactions. The training aimed to enable the patients to react only on relevant stimuli and use this strategy on their own in the future. The study showed that GMT is effective in improving executive functions in everyday life, as well as increasing the efficacy of problem solving which requires attention [9].

Cicerone and Wood’s study [11] is another example of executive functions rehabilitation. In this study, researchers used self-instructional procedure in rehabilitation of a patient with executive function disorder, caused by a prefrontal cortex traumatic injury. This patient had communication difficulties caused by impulsivity. Neuropsychological assessment detected a tendency for erroneous impulsive responses. During the study, this patient learned planning and problem solving, which involved developing a sequence of actions, their possible alternatives, and evaluation of the final result. This method involved three steps: from self-instructional procedure which involved overt verbalization to inner verbalization. After 8 weeks of applying strategies of self-regulation in everyday life, this patient succeeded in reducing off-task behaviours and problem-solving errors.

Rath and colleagues’ study [12] is an example of applying clinical practice for rehabilitation of behavioural/emotional self-regulation and executive functions. Researchers compared innovative approach of group therapy, involving emotional self-regulation and strategic thinking in problem solving, to traditional cognitive rehabilitation. Participants analysed their own impulsive reactions, wrote them down, as well as the events and sensations which preceded them. They next imagined how they could have changed the situation to avoid irrelevant emotional reactions. Moreover, participants applied strategies of emotional self-regulation, using a step-by-step program, involving different problem-solving strategies. Role playing and providing and receiving feedback were also key elements of this therapy. Researchers concluded that this method is more efficient in problem-solving, emotional self-regulation, and more realistic self-evaluation among participants, compared to traditional cognitive rehabilitation. All these improvements were preserved during the follow-up six months later [12].

Cicerone and Giacino’s study [13] described a rehabilitation of patients with difficulties in predicting results of their behaviour in unstructured social situations. These patients were unable to correct their social behaviour based on expected consequences of this behaviour, despite the satisfactory results of standardised neuropsychological tests. Rehabilitation involved training of the ability to predict the number of steps, necessary to complete the Tower of London task, receiving the feedback. The study showed that comparing predicted and actual results allowed patients to be more accurate in predicting the consequences of their behaviour in various social contexts. This study can be analysed as an example of rehabilitation of metacognitive function.

Another example of rehabilitation of metacognitive function is Ownsworth, McFarland, and Young’s study [14]. In this study, researchers described group therapy which focused on ameliorating self-regulation and self-awareness. Self-awareness was involved as a component of executive functions, in line with Stuss’s model. Based on this, researchers developed a rehabilitation program which involved methods of cognitive-behavioural therapy, cognitive rehabilitation and social skills learning, techniques of problem solving, relaxation, self-reflection, and role playing. A 16-week program of group training was aimed at ameliorating the skills of self-awareness and self-regulation and included a short description of mental functions (for example, memory), as well as obstacles people with brain injuries might face. During the discussion, all participants shared their difficulties and developed strategies to overcome them. After 16 weeks of this training of compensatory strategies and problem-solving, all participants showed a statistically significant increase in self-regulation and self-awareness [14].

We should note that the use of trainings based on cognitive strategies has a number of limitations. In our opinion, these restrictions may be caused by internal and external reasons. Internal reasons include gross violations of patients’ self-awareness, lack of motivation to follow instructions, difficulties associated with deficits of memory or thinking. By external reasons, we understand the difficulties of transferring skills to real life, which may be associated with the absence of relatives of the patient who could provide the necessary assistance in a situation of difficulty, or their demotivating attitude to the rehabilitation process, for example, overprotection.

This paper discusses the results of a group training of executive functions proposed by B. A. Wilson [15], the theoretical basis of which is the model of functioning of the frontal lobes described above [5]. This training, initially consisting of 9–10 group sessions, was adapted to the conditions of the Russian rehabilitation practice and shortened. The version developed by us includes three group and two individual sessions with each of the patients. New tasks relevant to patients and a functional trial were also added to the training. The made changes make it possible to apply the training not only for the rehabilitation of outpatient patients, but also for patients who are being treated in a hospital. This is especially important in the conditions of the Russian rehabilitation practice, where the standard period of stay in the hospital is 21 days. In addition, we believe that the possibility of applying the training of executive functions during hospitalization allows to start the rehabilitation process much earlier and reduce the risks of the formation of extensive secondary symptoms.

The present quasi-experiment aims to approbate Wilson’s executory functions training based on Stuss’s model, which involves executive and metacognitive functions [5,15]. The training was adapted to fit Russian clinical practice and included psychoeducation, instructing patients with regulatory deficits how to use various cognitive strategies, aiming to rehabilitate executive and metacognitive functions, and feedback on their success.

The hypotheses of the study were our assumptions that: (1) the effect of the training will be specific in relation to different domains; (2) in the course of the training, a decrease in executive deficit will be observed; (3) the specifics of the reduction of violations will be primarily related to the focus on the domains of executive thinking and metacognitive functions.

## 2. Participants and Methods

### 2.1. Participants

Sixteen participants (12 males, 4 females) took part in the study. All participants had executive function disorder different degrees of severity and did not have any impairments of speech and memory. All patients were administered with a neuropsychological assessment which evaluated the executive function disorder and neuropsychological status. All participants were informed of the aims of the study and the procedure. They were also given the opportunity to refuse to participate in the study at any time with no consequences.

The mean age of the participants was 48.1 y.o. (Median = 39, D = 424.25, SD = 20.6, age range 19–79 y.o.). The mean time after the first brain damage was diagnosed was 16.3 months (Median = 4.75 months, D = 557.73, SD = 23.6, ranged from 0.5 to 84 months), see Table 1 for details.

All subjects gave their informed consent for inclusion before they participated in the study. The study was conducted in accordance with the Declaration of Helsinki, and the protocol was approved by the Russian Psychological Society Ethics Committee on 17 October 2019 (the approval №110-20/2019).

### 2.2. Neuropsychological Assessment

All participants were administered with a full A.R. Luria assessment [16], as well as with a set of standardized tests evaluating the executive functions and general level of well-being twice: before and after the group training.

Table 2 illustrates methods used in the A.R. Luria assessment and criteria used to evaluate the impairments. Characteristics of these impairments were “negative symptoms”, which were evaluated on a scale from 0 (no symptoms), 1 (mild impairments or compensatory attempts) to 2 (severe impairments).

We also used the following standardized tests: Colour–Word Interference Test (CWIT, total corrected errors and total uncorrected errors were evaluated, as well as the time spent on this task); Raven’s Progressive Matrices (all types of errors were evaluated); Frontal assessment battery (FAB, we evaluated how successful the participant was in the following tasks: Conceptualization, Lexical fluency, Motor series “Luria” test, Conflicting instructions, Inhibitory control, Prehension behaviour) and European brain injury questionnaire (EBIQ; the total score was evaluated).

### 2.3. Statistical Analysis

Data analysis was conducted for each diagnostic method we used: we compared the data collected before and after the training.

Statistical analysis was based on non-parametric statistics: Wilcoxon signed-rank test and the sign test for the tasks involving a three-point scale (0-1-2). Differences between scores were considered statistically significant with a *p*-value < 0.05. Statistical analysis was performed using IBM Statistical Package for the Social Sciences 22 (IBM Corp., Redmont, VA, USA).

### 2.4. Rehabilitation Method

We used a modified group training of executive functions, described in The Brain Injury Rehabilitation Workbook [15]. The modification was aimed at rehabilitating the domains of executive and metacognitive functions.

The group training was conducted from November 2019 to March 2020 at the Moscow region rehabilitation centre “Three sisters” (“Tri sestry”). Sixteen participants took part in this training (four experimental groups; four participants in each group).

The training program involved three weekly group training sessions, dedicated to mental health education in executive functions in line with Stuss’ model and to the potential behavioural problems which can develop because of the executive and metacognitive functions impairments. Moreover, during this group training all participants completed specific exercises which aimed at both executive and metacognitive functions, learned-, and applied cognitive strategies suggested by the authors.

Below we describe the main cognitive strategies which aim to ameliorate the executive function domains, suggested by the authors of the training, Wilson and colleagues [15].

Stop/Think technique. This cognitive strategy allows to thoroughly plan actions before performing them. The authors of the training suggest using this strategy for the executive function’s rehabilitation. This strategy aims at supressing impulsive reactions, ameliorating of planning and decision-making processes [15]. It can be used as part of the Goal Management Framework (GMF) to facilitate planning processes, decision-making, goal achievement and problem solving. During the training, participants were asked to choose between different options and put together the order of steps necessary to build a house. They were also asked to suggest their own ideas on the steps necessary to build a house and suggest how to order them.

GMF is a scheme which involves six steps for everyday-life problem-solving. The authors suggest using this scheme as a part of the executory function’s rehabilitation [15]. For a more efficient choice of alternative problem-solving options, pros and cons strategy is involved in this technique. Pros and cons strategy allows to analyse all advantages and disadvantages of these alternative options. Participants were asked to choose the most appropriate transport to travel from Moscow to Saint-Petersburg, analysing the advantages and disadvantages of all possible options.

The Zoom In/Out technique allows patients to analyse the problem from different points of view and taking time into account. For instance, patients were asked to imagine long-term consequences (a week, a month, a year, three years) of a hypothetical decision to study Chinese language. This technique allows to solve problems which involve too-abstract or too-concrete thinking, allowing to reach the necessary abstraction level. On the other hand, it allows to increase the level of self-awareness and sensitivity to the aims and positions of other people. This enables to use this technique for rehabilitation of executive and metacognitive functions.

Moreover, the authors of the training advise to provide patients with the feedback and conduct behavioural experiments for metacognitive function rehabilitation.

In conclusion, to strengthen the achieved effects participants were completing different exercises, home tasks, taking part in group discussions, aiming to increase awareness on the role of executive functions in everyday life and strengthen the application of cognitive strategies described above. For a more successful application of theoretical knowledge in real life participants were also suggested to complete a practical task: a behavioural experiment focusing on autonomous shopping in a special “shop” in the rehabilitation centre’s ergonomic zone.

## 3. Results

Statistical analysis was conducted to compare the tests’ scores and their parameters before and after the training, and to evaluate the dynamics of the executive functions caused by the training sessions. Statistical analysis was based on total scores of all methods we conducted and revealed significant differences in the following tasks: *understanding of thematic pictures*, *p* = 0.011, *counting,* *p* = 0.039, and the CWIT’s parameter *total uncorrected errors,* *p* = 0.028 (see Table 3).

Table 4 illustrates the results of statistical analysis of methods described above. We can see that the differences in these methods before and after the training are significantly different.

**Figure 1:**
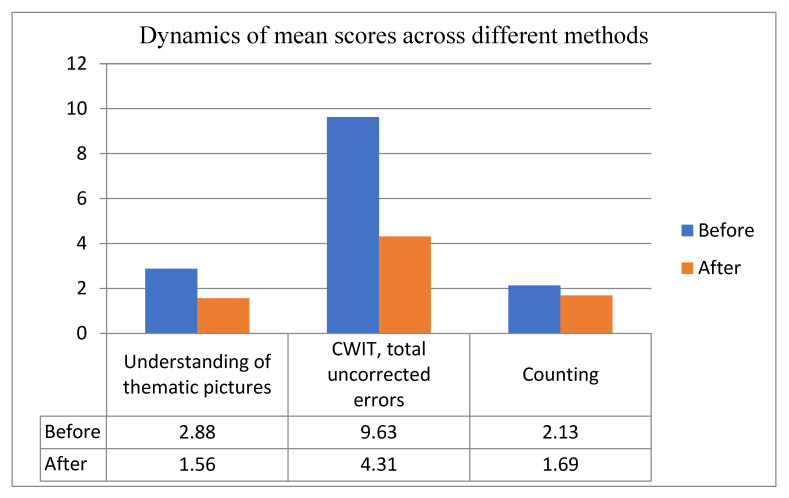
Dynamics of mean scores of methods which differed significantly before and after the training sessions.

Figure 1 illustrates the dynamics of mean scores of the above-mentioned methods. Total score indicates the mean number of errors.

We can see that in all three cases the mean score was significantly lower, which indicates positive dynamics.

Methods used to evaluate executive functions in line with A.R. Luria’s system also revealed some significant differences before and after the training (see Table 5). For instance, parameter *unable to understand the meaning of the story* (Retelling a story method) differed significantly before and after the training (*p* = 0.038). *Fragmentation of the analysis* and *inability to describe the narrative* (parameters of the Understanding of thematic pictures method) changed significantly after the training (*p* = 0.034 and *p* = 0.023 respectively), which can be related to the improvement of executive functions.

**Figure 2:**
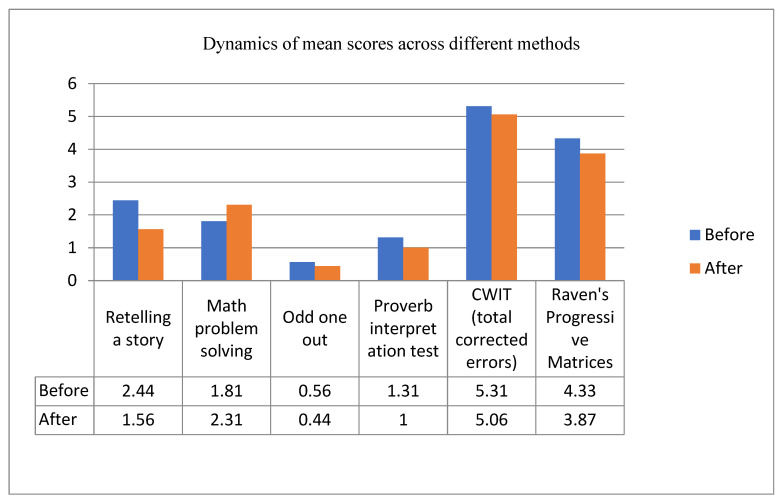
Dynamics of mean scores of methods which did not differ significantly before and after the training.

Although other methods and parameters did not differ significantly before and after the training, there was a tendency for positive dynamics. For instance, Figure 2 illustrates dynamics of mean scores of other methods before and after the training.

During the final testing we detected differences in the mean score in the following methods: Retelling a story, Odd one out, Proverb interpretation test, CWIT (total corrected errors score) and Raven’s Progressive Matrices. As already mentioned, these differences were not statistically significant (*p* > 0.05). However, it should be mentioned that a decreased mean score indicates that less errors were made, and participants were more successful in their performance. However, with one exception: math problem solving was worse after the training. It should be mentioned that the training did not aim to improve math skills. We may assume that patients were more accurate and less impulsive in math problem solving, however, because of the cognitive load they were unable to solve math problems efficiently.

CWIT test showed a decreased total corrected error score after the training. This indicates that patients were more effective in controlling irrelevant verbal responses, as well as correcting their own errors more efficiently. Mean time spent on this test also decreased from 78.28 s before the training to 68.05 s after the training.

Lower mean scores of Raven’s Progressive Matrices also indicate a positive dynamics of rehabilitation process. More specifically: lower rate of impulsive responses and better orientation in the task after the training.

Comparing EBIQ results before and after the training, we also detected that total score decreased (from 97.1 before the training to 91.2 after the training). This indicates that patients’ wellbeing increased, while perceived evaluation of difficulties decreased. However, these differences were not statistically significant (*p* > 0.05).

**Figure 3:**
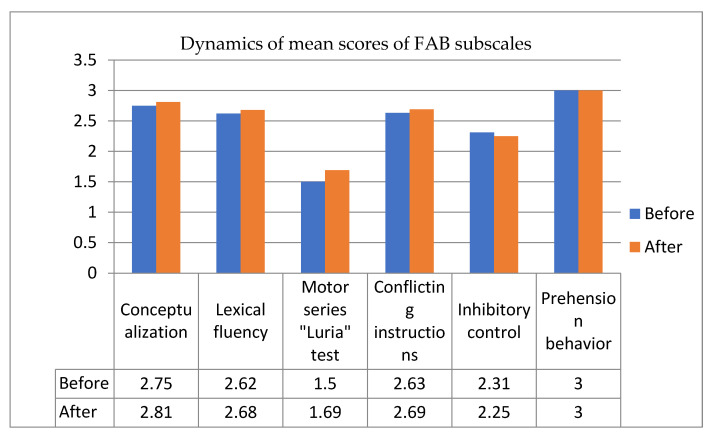
Dynamics of mean scores of FAB subscales before and after the training (did not differ significantly).

Positive dynamics was detected across all parameters of FAB, excluding inhibitory control parameter (see Figure 3). Increased mean score in this case indicates a more successful performance. However, decreased inhibitory control parameter indicates that positive changes were not stable across synthetic tests.

## 4. Discussion

In the present study we approbated a group training, developed by B. Wilson and colleagues [15]. The training was based on Stuss’s model of frontal lobes. Theoretical background of this training aimed at improving the domains of executive and metacognitive functions, which can affect patients’ everyday life and communication.

In line with data obtained in neuropsychological assessment before and after the rehabilitation process, patients demonstrated positive dynamics across most measures.

Understanding of thematic pictures and CWIT (total uncorrected errors score) methods were most sensitive to the training effects. We may assume that these results indicate an improvement in executive and metacognitive functions. Executive function is traditionally related to self-control, while metacognitive function is traditionally related to self-awareness and situation awareness. Probably, cognitive strategies Stop/Think and Zoom In/Out allowed our patients to evaluate their CWIT performance more critically and correct their own errors, as well as analyse thematic pictures more accurately and from different points of view. In favour of this assumption, we found positive dynamics in *fragmentation of the analysis* and *inability to describe the narrative* (parameters of the Understanding of thematic pictures method).

We also found significant differences in one subscale of Retelling a story method: unable to understand the meaning of the story. Probably, this is related to the fact that our patients were able to retain the line of the story better, analyse the narrative and be more accurate with significant details. This positive dynamic can be related to improvement of executive functions. For instance, executive functions contribute to programming and control (in this case—in story telling), while metacognitive functions can be related to better orientation in the story line.

Statistically significant improvement in Counting measure may also suggest an improvement in executive functions domain, associated with programming (in this case–programming of a sequence of math operations) and control.

These results may indicate the effectiveness of the training proposed by the authors [15], even in its shortened version adapted to the conditions of Russian clinical practice.

Although at this stage we discuss positive dynamics across different measures and may assume that it is related to improvement of executive functions, we are also aware of limitations of this study.

Firstly, we did not include a control group in this study. A control group allows to reject alternative explanations of the results we obtained during the second neuropsychological assessment. This limitation is caused by Covid-19 pandemics, which forced us to terminate the study earlier. However, we decided to share the data we obtained despite this limitation and publish the obtained results.

The second aspect that needs to be taken into account is the small sample size. The heterogeneous composition of the group could lead to an increase in the size of the variance. Conducting a study on a larger number of participants will help reduce the impact of this factor and obtain more reliable data.

Thirdly, the standard rehabilitation program in Russia is typically 2-weeks long. It allows only few training sessions which are not enough to develop necessary skills and knowledge. However, as this study shows, even such a short-term training can improve executive functions.

Finally, it is not obvious how to link methods we used with specific domains of executive functions. As all mental functions, executive functions are closely related to each other in one functional system.

## 5. Conclusions

In present study we demonstrated how training of executive functions can be applied in Russian clinical practice. The procedure of training and training tasks demonstrated their adequacy for rehabilitation of patients with executive and metacognitive functions impairments. Even a small amount of training sessions we conducted during the rehabilitation process improved patients’ cognitive skills and performance.

Positive dynamics were observed across the majority of diagnostic methods we used, and statistically significant results were obtained across the following methods: counting, understanding of thematic pictures, and CWIT (total uncorrected errors score).

This data allows to assume that there is positive dynamics in executive and metacognitive functions.

Further studies should consider and overcome the limitations of present study. More specifically: future studies should include both experimental and control groups, larger sample size and more diverse and improved training program.

## Figures and Tables

**Table 1:** Participants’ data.

Participant’s ID	Gender	Age	Diagnosis	Time Since Diagnosis
N1	F	72	Subarachnoid and intraventricular hemorrhage. Solid-cystic mass in the left temporal lobe.	9 months
N2	M	40	Ischemic stroke in the middle cerebral artery.	9 months
N3	M	54	Repeated cerebrovascular accident: ischemic accident in the middle cerebral artery.	4 months
N4	F	35	Traumatic brain injury with primary diffuse axonal injury.	34 months
N5	M	73	Subocclusion of the right internal carotid artery, acute ischemic cerebrovascular syndrome.	0.5 months
N6	M	36	Ischemic stroke in the right middle cerebral artery and posterior cerebral artery, occlusion of the right internal carotid artery.	3.5 months
N7	M	19	Severe closed traumatic brain injury. Post-traumatic glial and cystic changes in the brain.	5.5 months
N8	F	65	Cerebral infarction.	4 months
N9	M	70	Intracerebral haemorrhage in the left hemisphere with ventricular extension.	1 month
N10	M	38	Encephalomyelitis.	84 months
N11	M	33	Acute traumatic brain injury, severe cerebral contusion, diffuse axonal brain injury, craniotomy in the right frontal parietal lobe, intracranial haemorrhage.	12 months
N12	M	72	Cerebral infarction in the left middle cerebral artery.	1.5 months
N13	F	35	Acute ischemic cerebrovascular syndrome in the right middle cerebral artery.	36 months
N14	M	25	Closed traumatic brain injury, severe cerebral contusion, diffuse axonal brain injury.	52 months
N15	M	79	Acute ischemic cerebrovascular syndrome in the basilar artery and posterior brain arteries.	1.5 months
N16	M	24	Closed traumatic brain injury.	3 months

**Table 2:** List of methods used to evaluate executive functions in line with A.R. Luria’s system, as well as the criteria used to evaluate the impairments.

**Method**	**Estimated Parameters**
Retelling a story	General understanding of a story with lack of details Unable to understand the meaning of the story Derailment to side associations Reduced selectivity
Math problem solving	Unable to understand the conditions of the task Difficulties in development of the program Inhibitory control impairment: unable to control primary impulsive operations Unable to compare the results of the program with the conditions of the task Difficulties in switching when the algorithm of the task changes
Counting	Unable to follow the program or to verbalize it Inhibitory control decrease Impairment of counting below ten Impairment of counting within ten
Understanding of thematic pictures	Fragmentation of the analysis Equiprobable activation of different hypothesis Decline of the generalization level Derailment to side associations Formal description of the elements of thematic pictures Inability to describe the narrative
Odd one out	Decline of the generalization level
Proverb interpretation test	Unable to understand the figurative interpretations of the proverbs Unable to verbalize the meaning of the proverb Derailment to side associations Perseverations in thinking

**Table 3:** Total scores across methods which differed significantly before and after the training sessions (*p* ≤ 0.05).

Method	Measure	Number of Participants	Min	Max	Median	Mean Score	SD
Understanding of thematic pictures	Before	16	0	7	2	2.88	2.53
	After	16	0	5	1	1.56	1.46
CWIT (total uncorrected errors)	Before	16	0	27	10	9.63	8.54
	After	16	0	18	3	4.31	4.54
Counting	Before	16	0	8	2.5	2.13	2.22
	After	16	0	8	0	1.69	2.55

**Table 4:** Wilcoxon signed ranks test and Sign test results across methods which differed significantly before and after the training sessions (*p* ≤ 0.05).

Statistical Criteria	Method	Parameter	N	Mean Rank	Sum of Ranks	Z-Score	*p*-Value
Wilcoxon Signed Ranks Test	Understanding of thematic pictures	Negative ranks	10 ^a^	7.10	71.0		
		Positive ranks	2 ^b^	3.50	7.00	−2.547	0.011
		Ties	4 ^c^				
		Total	16				
	CWIT (total uncorrected errors)	Negative Ranks	12 ^a^	9.21	110.5		
		Positive Ranks	4 ^b^	6.38	25.5	−2.201	0.028
		Ties	0 ^c^				
		Total	16				
Sign Test	Counting	Negative Differences	8				0.039
		Positive Differences	1				
		Ties	7				
		Total	16				

^a^ Number of cases in which the error parameter After the training is less than Before the training; ^b^ Number of cases in which the error parameter After the training is bigger than Before the training; ^c^ Number of cases in which the error parameter After the training equals the number Before the training.

**Table 5:** Results of the statistical analysis of subscales which differed significantly before and after the training (*p* ≤ 0.05).

Statistical Criteria	Method	Parameter	N	Mean Rank	Sum of Ranks	Z-Score	*p*-Value
Wilcoxon Signed Ranks Test	Missing the meaning of the story («Retelling a story»)	Negative ranks	5 ^a^	3.00	15.0		
		Positive ranks	0 ^b^	0.00	0.00	−2.070	0.038
		Ties	11 ^c^				
		Total	16				
	Analysis fragmentation («Understanding of thematic pictures»)	Negative ranks	7 ^a^	4.50	31.5		
		Positive ranks	1 ^b^	4.50	4.50	−2.121	0.034
		Ties	8 ^c^				
		Total	16				
	Being unable to describe the story («Understanding of thematic pictures»)	Negative ranks	6 ^a^	3.50	21.0	−2.271	0.023
		Positive ranks	0 ^b^	0.00	0.00		
		Ties	10 ^c^				
		Total	16				

^a^ Number of cases in which the error parameter After the training is less than Before the training; ^b^ Number of cases in which the error parameter After the training is bigger than Before the training; ^c^ Number of cases in which the error parameter After the training equals the number Before the training.
